# Supplementary material for: Cost-effectiveness of comparative survey designs for helminth control programs: Post-hoc cost analysis and modelling of the Kenyan national school-based deworming program
Source: PLoS Negl Trop Dis. 2024 Dec 2;18(12):e0011583. doi: 10.1371/journal.pntd.0011583 (PMC11666021; doi:10.1371/journal.pntd.0011583)
Supplement: S1 Table — (DOCX) [file pntd.0011583.s001.docx]

**S1 Table.** Itemized cost for individual survey components, totaled for a survey comprising of 200 schools and 100 students per school.

| Stage | Activity/item class 1 | Item class 2 | Type or description | Total USD schools (200) samples (100) | Notes |
| --- | --- | --- | --- | --- | --- |
| Base-training | Travel | Training travel reimbursement | Travel + incidentals | 5980 | Fixed cost |
| Base-training | Venue hire | Conference hall hire | Single payment | 1104 | Fixed cost |
| Base-training | Lab equipment | Equipment hire | One off (per day) | 9200 | Fixed cost |
| Base-training | Venue hire | Lab space | One off (per day) | 920 | Fixed cost |
| Base-training | Salary | Lab trainers | Allowance | 1840 | Fixed cost |
| Base-training | Salary | Trainers’ salary | Daily salary | 1840 | Fixed cost |
| Base-training | Incidentals | Data and process trainer allowance | Allowance | 920 | Fixed cost |
| Base-training | Salary | Data and process trainer salary | Daily salary (in kind) | 1840 | Fixed cost |
| Base-training | Venue hire | Hire of training facility | Rental | 73.6 | Fixed cost |
| Base-training | Incidentals | Refreshments + lunch | Food/beverage | 23 | Fixed cost |
| Base-training | Lab equipment | Consumables (training) | Disposable equipment | 460 | Fixed cost |
| Sampling | Travel | Fuel costs - near | Average | 2906.28 | Multiplied per school or per sample |
| Sampling | Travel | Fuel costs - far | Average | 388.4608 | Multiplied per school or per sample |
| Sampling | Salary | Field staff wage | Field supervisor | 50232 | Multiplied per school or per sample |
| Sampling | Salary | Field staff wage | Laboratory technologist | 33488 | Multiplied per school or per sample |
| Sampling | Salary | Field staff wage | Laboratory assistant* often local | 19320 | Multiplied per school or per sample |
| Sampling | Salary | Field staff wage | Data manager | 41952 | Multiplied per school or per sample |
| Sampling | Salary | Field staff wage | Data clerk | 19320 | Multiplied per school or per sample |
| Sampling | Salary | Field staff wage | Driver | 19320 | Multiplied per school or per sample |
| Sampling | Salary | Field staff wage | Cleaner | 11592 | Multiplied per school or per sample |
| Sampling | Salary | Local security | For security risk areas | 1104 | Multiplied per school or per sample |
| Sampling | Venue hire | Laboratory space | Space hire | 9200 | Multiplied per school or per sample |
| Sampling | Incidentals | Community mobilization materials | Per school, multiple days | 49680 | Multiplied per school or per sample |
| Sampling | Lab equipment | Labels | Packet (400 labels) 1 per school | 506 | Multiplied per school or per sample |
| Sampling | Lab equipment | Gloves | Packet (100) 2 schools | 1380 | Multiplied per school or per sample |
| Sampling | Lab equipment | Slide box | 2.5 boxes per school (100 slides) per school | 2208 | Multiplied per school or per sample |
| Sampling | Lab equipment | Cooler packs | 8 cooler boxes (used for all schools) 2 Per school (1 big and 1 small) | 588.8 | Multiplied per school or per sample |
| Sampling | Lab equipment | Cooler packs |  | 368 | Multiplied per school or per sample |
| Sampling | Lab equipment | Microscope | 2 needed per team (re-usable) (school) | 7360 | Multiplied per school or per sample |
| Sampling | Lab equipment | Forceps | 2 per team (re-usable) | 18.4 | Multiplied per school or per sample |
| Sampling | Lab equipment | Marker pens - pkts | 2 pens per school | 368 | Multiplied per school or per sample |
| Sampling | Lab equipment | Glycerol(liters) | 5 liters enough for whole exercise | 46 | Multiplied per school or per sample |
| Sampling | Lab equipment | Malachite green | 1 packet (100 grams) enough for whole exercise | 23 | Multiplied per school or per sample |
| Sampling | Lab equipment | Templates | 100 pieces per school | 92000 | Multiplied per school or per sample |
| Sampling | Lab equipment | Stool sieves | 50 pieces per school | 92000 | Multiplied per school or per sample |
| Sampling | Lab equipment | Buckets | 2 per school | 368 | Multiplied per school or per sample |
| Sampling | Lab equipment | Basin | 2 per school | 368 | Multiplied per school or per sample |
| Sampling | Lab equipment | Tally counters | 2 per school | 3680 | Multiplied per school or per sample |
| Sampling | Lab equipment | Sharps Containers | 1 per school | 920 | Multiplied per school or per sample |
| Sampling | Lab equipment | Slides: stool(100.box) | 2 packets per school (100 per packet) | 1840 | Multiplied per school or per sample |
| Sampling | Lab equipment | Stool poly pots(200/pkt) | 1 packet per school | 9200 | Multiplied per school or per sample |
| Sampling | Lab equipment | Wooden spatula 100/box | 2 packets per school | 736 | Multiplied per school or per sample |
| Sampling | Lab equipment | Slides Urine - 100/box | 2 packets per school | 736 | Multiplied per school or per sample |
| Sampling | Lab equipment | Urine chambers | 1 packet per school (100 chambers) | 4600 | Multiplied per school or per sample |
| Sampling | Lab equipment | Filters boxes of 100 | 1 packet per school (100 filters) | 3680 | Multiplied per school or per sample |
| Sampling | Lab equipment | Syringes - boxes | 100 pieces per school | 3680 | Multiplied per school or per sample |
| Sampling | Lab equipment | Urine containers | 1 packet per school (about 200 pieces) | 9200 | Multiplied per school or per sample |
| Sampling | Lab equipment | Toilet paper - bales | 5 pieces per school | 460 | Multiplied per school or per sample |
| Sampling | Lab equipment | Disinfectant:5litres | 10 liters for whole exercise | 3680 | Multiplied per school or per sample |
| Sampling | Lab equipment | Hand soap (Dettol) | 1 unit per school | 92 | Multiplied per school or per sample |
| Sampling | Lab equipment | Detergent(1kg) | 0.5 kg per school | 276 | Multiplied per school or per sample |
| Sampling | Lab equipment | Towels | 2 per school | 1472 | Multiplied per school or per sample |
| Sampling | Lab equipment | Toothbrush | 2 pieces per school | 184 | Multiplied per school or per sample |
| Sampling | Lab equipment | Biohazard bags | 1 piece per school | 184 | Multiplied per school or per sample |
| Sampling | Lab equipment | Bin bags | 1 piece per school | 147.2 | Multiplied per school or per sample |
| Sampling | Lab equipment | Bins | 1 piece per school | 920 | Multiplied per school or per sample |
| Sampling | Lab equipment | Newspaper | 0.5 kg per school | 46 | Multiplied per school or per sample |
| Sampling | Lab equipment | Paper towels - rolls | 1 roll per school | 276 | Multiplied per school or per sample |
| Sampling | Lab equipment | Biros(box) | 2 pens per school | 73.6 | Multiplied per school or per sample |
| Sampling | Lab equipment | Pencils(box) | 2 pencil per school | 73.6 | Multiplied per school or per sample |
| Sampling | Lab equipment | Lab coats (personal protective equipment) | 10 pieces for survey | 184 | Multiplied per school or per sample |
| Sampling | Lab equipment | Sanitizers | 0.5 liter per school | 552 | Multiplied per school or per sample |
| Sampling | Lab equipment | Masks | 1 box per school | 920 | Multiplied per school or per sample |
| Sampling | Survey equipment | Mobile phone | 1 per team | 276 | Multiplied per school or per sample |
| Sampling | Survey equipment | Mobile phone data (bundles) | 1000 mb per school | 920 | Multiplied per school or per sample |
